# Effect of Surface Finishing on the Corrosion Resistivity of 3D Printed M300 Steel

**DOI:** 10.3390/ma17246047

**Published:** 2024-12-10

**Authors:** Krzysztof Żaba, Krzysztof Szymański, Maciej Balcerzak, Ilona Różycka, Łukasz Kuczek, Piotr Żabiński

**Affiliations:** 1 Department of Metal Working and Physical Metallurgy of Non-Ferrous Metals, Faculty of Non-Ferrous Metals, AGH University of Krakow, al. Adama Mickiewicza 30, 30-059 Cracow, Poland; krzyzaba@agh.edu.pl (K.Ż.); kszymanski@student.agh.edu.pl (K.S.); balcerzak@agh.edu.pl (M.B.); lukasz.kuczek@agh.edu.pl (Ł.K.); 2 Department of Materials Science and Engineering of Non-Ferrous Metals, Faculty of Non-Ferrous Metals, AGH University of Krakow, al. Adama Mickiewicza 30, 30-059 Cracow, Poland; rozycka@agh.edu.pl; 3 Department of Physical Chemistry and Metallurgy of Non-Ferrous Metals, Faculty of Non-Ferrous Metals, AGH University of Krakow, al. Adama Mickiewicza 30, 30-059 Cracow, Poland

**Keywords:** 3D printing, DMLS method, M300 steel, corrosion, surface finishing

## Abstract

The purpose of this study was to investigate the influence of synthesis parameters and surface finish on the corrosion of DMLS-printed M300 steel components and to evaluate their applicability in corrosive environments. In order to assess the influence of the corrosive environment, potentiodynamic and long-term corrosion tests were carried out in this study, together with microscopic and EDS studies on 3D-printed M300 steel samples synthetized using the DMLS method with different laser powers. The results show that DMLS-produced M300 steel is vulnerable to corrosion in corrosive environments. The effect of the laser power used on the corrosion resistance was also demonstrated, which generally decreases with increasing laser power. This study confirms the influence of the surface condition of the components on the corrosion phenomenon. Despite the higher corrosion resistance of unpolished components, they lose mass to a higher degree in a corrosive environment. This study also shows the influence of temperature on the corrosion phenomena occurring, demonstrating its negative effect. This study also presents the microstructure of the surface of the samples after the tests, showing the degradation of the surface due to corrosive actions. The analysis of the test results suggests the protection of M300 steel components produced using the DMLS method for the case of operation in corrosive environments.

## 1. Introduction

Additive manufacturing (AM) technologies encompass a range of modern production methods aimed at automating the layer-by-layer fabrication of objects based on suitably prepared CAD models, while minimizing the use of traditional machining tools [1,2]. AM has been identified as one of nine cutting-edge technologies that play a significant role in the fourth industrial revolution. This advanced and widely adopted manufacturing technique is particularly beneficial for producing small components, complex geometrical products, and custom-made elements, all while optimizing structure and minimizing material waste [3,4,5,6].

Additive manufacturing processes can be categorized into several groups, with two predominant methods: Powder Bed Fusion (PBF) [7] and Directed Energy Deposition (DED) [8]. In DED, processes such as 3D laser cladding, Laser Metal Deposition (LMD), and Laser Engineered Net Shaping (LENS) utilize an integrated powder feeder and an energy source, which may be either a laser or an electron beam. These components work in tandem to produce a molten layer of material on a substrate surface. Conversely, PBF includes methods such as Selective Laser Sintering (SLS), Selective Laser Melting (SLM) [9], Electron Beam Melting (EBM) [10], and Selective Heat Sintering (SHS) [11]. In this technology, a thin layer of powder is meticulously applied to a substrate, and the energy source—most commonly a laser or electron beam—fuses the powder particles into a cohesive layer. SLM stands out among other PBF technologies due to its capability to achieve nearly 100% density in final products, making it one of the most efficient methods for processing metallic materials [12,13,14].

In the context of DED, its popularity is rising primarily due to its ability to not only produce new components but also repair damaged parts. The feedstock material, which can be either powder or wire, is delivered through the printing head, where the material and energy source are integrated [15,16]. Powder-based systems in DED are known as Laser Metal Deposition machines [17], while wire-based processes, such as Electron Beam Additive Manufacturing (EBAM) [18] and Wire Arc Additive Manufacturing (WAAM) [19], offer alternative approaches within this technology. PBF is one of the most frequently utilized AM methods, finding applications in industries such as aerospace, medicine, and the automotive and tooling industries. This process is based on applying a thin layer of powder onto a build plate, followed by melting or sintering the powder layer by layer using energy from a laser or electron beam. This technology can be divided into SLM, SLS, and EBM, each offering unique advantages and disadvantages depending on specific applications. When comparing DED and PBF technologies, DED provides greater flexibility in terms of heat source and feedstock material selection, allowing for the production of larger components and the repair of existing parts [20]. In contrast, PBF offers higher print precision and superior mechanical properties for products, enabling the concurrent production of multiple components in a single manufacturing cycle. PBF systems, such as Laser Powder Bed Fusion (LPBF) and Electron Beam Powder Bed Fusion (EB-PBF), differ in the energy sources employed, which impacts the specific properties of the final products [21,22,23]. LPBF provides greater dimensional accuracy and smoother surfaces, making it the preferred technology for many advanced engineering applications.

Maraging steels are particularly well-suited for SLM due to two primary reasons. Firstly, their martensitic structure necessitates rapid cooling from the austenitic region to temperatures below the martensite start point. In the SLM process, the small size of the melt pool typically results in extremely high cooling rates. Secondly, maraging steels have thus far found their main applications in the aerospace and tooling industries, where their high costs limit broader use in production. These sectors often require complex geometries for the manufactured components, and the SLM process effectively meets these demands in small production runs [24,25,26].

Maraging steels are highly regarded in the aerospace industry, where they are used in the construction of components such as aircraft fuselages, rocket housings, bearings, and drive and fan shafts. Due to their low reflectivity and excellent welding properties, maraging steel powder is ideal for processing via Selective Laser Melting (SLM). The carbon content in maraging steel is minimal (approximately 0.03%), which translates to outstanding weldability and material strength. The SLM process involves various parameters, including laser spot diameter, laser power, path spacing, scanning patterns, scanning speed, and layer thickness, all of which significantly influence the mechanical and physical properties of the final product. A critical challenge in additive manufacturing is the mechanical anisotropy observed between the direction normal to the layer and the build plane. Maraging steels exhibit a combination of high strength and exceptional fracture resistance. This property is achieved through a specific microstructure consisting of a cubic martensitic matrix, containing nanometric particles of various intermetallic phases [26,27,28]. These particles are generated during heat treatment, conducted at temperatures below the austenite transformation point.

The production process for maraging steel involves several stages, including block casting, hot rolling, solution heat treatment, quenching, machining, and final aging. To manufacture small, custom, high-precision components from metallic powder, Selective Laser Melting (SLM) has become a key area of research. The production of maraging steel using SLM results in significantly higher yield strength and tensile strength compared to materials processed through traditional manufacturing methods. This can largely be attributed to the very fine microstructure formed during SLM, where the elongated grains arise from cellular solidification [29,30]. Furthermore, this process may lead to precipitation hardening, as during the application of successive layers, the underlying material is partially remelted or heated to high temperatures, causing the material in one layer to be within the heat-affected zone of the layer above.

Molds frequently come into contact with coolants during use, making corrosion resistance a key factor alongside mechanical and thermal properties. The M300 material is a mild corrosion-resistant steel suitable for NaCl-containing aqueous environments at room temperature, with pitting being the primary form of corrosion observed [31]. The presence of pores in the components diminishes their corrosion resistance, and larger defects increase susceptibility to corrosion. Anisotropy in the microstructure, which depends on the build direction [32], can result in varying electrochemical behaviors in different orientations. The corrosion performance of M300 can be enhanced through the HIP process, which reduces defects and increases the amount of austenite [33]. Comparatively, age-hardened M300 steel has poorer corrosion resistance than solution-annealed M300, likely due to particle precipitation [34,35]. Solution annealing at high temperatures removes segregated sub-grain cells containing Ni and Co and forms Ti-rich precipitates, thereby degrading the corrosion properties of the as-built M300 components [36]. According to Tonolini et al. [37] M300 samples demonstrate better corrosion resistance than cold-rolled samples, likely due to their finer microstructure and greater austenite volume fraction.

This study examines the electrochemical performance of M300 steel components produced through 3D printing. Components with two types of surface finishes were subjected to oxygen-saturated water containing chlorides at both room and elevated temperatures. The corrosion rate was assessed using electrochemical polarization measurements and the weight loss method. Additionally, the microstructure of the corroded parts was analyzed to gain insights into the corrosion mechanisms and the influence of surface finishing and microstructure at the interface.

## 2. Materials and Methods

**Figure 1:**
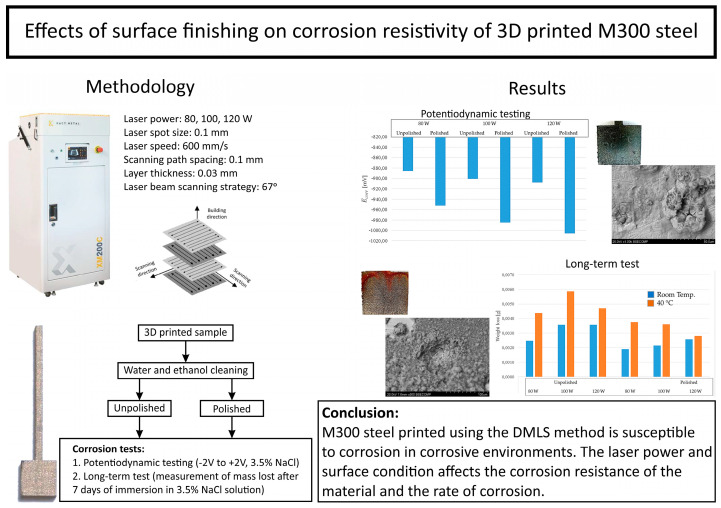
Introductory graphical abstract for the research processes.

The Introductory graphical abstract for the research processes is shown in Figure 1.

### 2.1. Material

**Figure 2:**
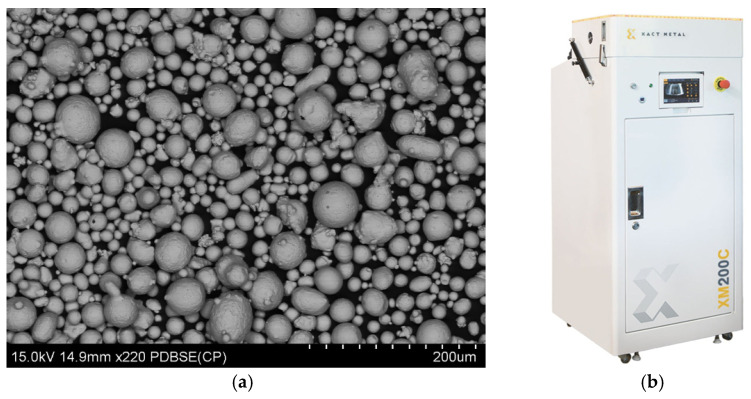
Xact Metal XM200C 3D Printer (**a**), SEM image of M300 steel powder (**b**).

The material for the LPBF method of 3D printing, used to produce specimens designed for corrosion testing, was gas-atomized martensitic precipitation-hardened maraging tool steel M300 (Steel 1.2709) spherical powder, characterized by a particle size distribution in the range of 20 to 90 μm (Figure 2a), supplied by Praxair Surface Technologies (Indianapolis, IN, USA). The chemical composition of the maraging steel M300 is presented in Table 1. The main alloying elements of this steel are nickel, cobalt, and molybdenum.

The specimens were 3D printed using the XM200C device (Figure 2b) (Xact Metal, State College, PA, USA).

Identical samples were printed for the tests using the same process settings except for the laser power, which for individual batches was 80 W, 100 W, and 120 W. The process parameters are presented in Table 2.

### 2.2. Methods

**Figure 3:**
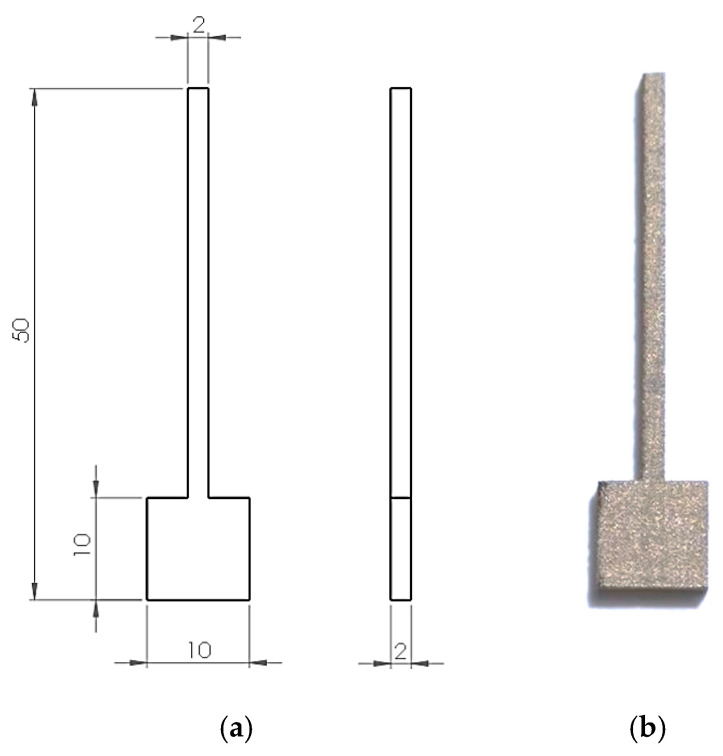
A 3D model (**a**) and an exemplary sample made using the DMLS method (**b**).

The 3D model of the test sample is shown in Figure 3a and an exemplary sample made using the DMLS method is shown in Figure 3b. The lower area of the sample, with dimensions of 10 mm by 10 mm and a thickness of 2 mm, was tested.

After 3D printing, all samples were cleaned using distilled water then ethanol and were then air dried at room temperature. Additionally, some of them were subjected to a mechanical polishing process.

The samples were subjected to two types of corrosion tests: potentiodynamic testing, and long-term corrosion testing, carried out by measuring the mass loss of the samples after 7 days of immersion in 3.5% NaCl solution.

The potentiodynamic study was performed on both unpolished and polished samples produced with different laser power using a BioLogic SP-200 potentiostat (Bio-Logic SAS, Claix, France) and analyzed using EC-Lab V10.44 computer software. This study used a potential sweep in the range of −2 V to +2 V relative to the open cell potential (OCP) at a rate of 0.01 V/s. The measurement was performed in a vessel filled with a 3.5% NaCl solution in which a calomel electrode was placed as a reference electrode. A platinum mesh was used as the counter electrode, while the samples under study were the working electrode. The long-term mass loss measurement was performed by weighing the samples on an analytical balance (with a measurement accuracy of 0.00001 g) and then immersing them in a 3.5% NaCl solution for 7 days. Half of the samples were placed in an incubator at 40 °C, and the other half were left at room temperature. After removing the samples from the containers in which they were tested, they were thoroughly rinsed with distilled water and ethyl alcohol and dried. Each sample was then re-weighed. The microstructure of the samples was observed both from the surface and on the cross-section of the metallographic specimens. The samples for microstructural analysis were embedded in Struers epoxy resin (Copenhagen, Denmark). Grinding was carried out according to Struers recommendations using SiC abrasive papers of gradation #220, #500, #800, #1200, #2000, and #4000. The samples were then polished using MD MOL polishing discs using a 3 μm diamond paste. The finishing stage was carried out using a silicon dioxide suspension, OP-S (Struers).

The analysis of the microstructure of the samples was carried out using a scanning electron microscope SU 70 (Hitachi Ltd., Tokyo, Japan). The analysis of the chemical composition in micro-areas was performed using the energy dispersive spectroscopy (EDS) method.

## 3. Research Results and Discussion

### 3.1. Description of the Tested Material

**Figure 4:**
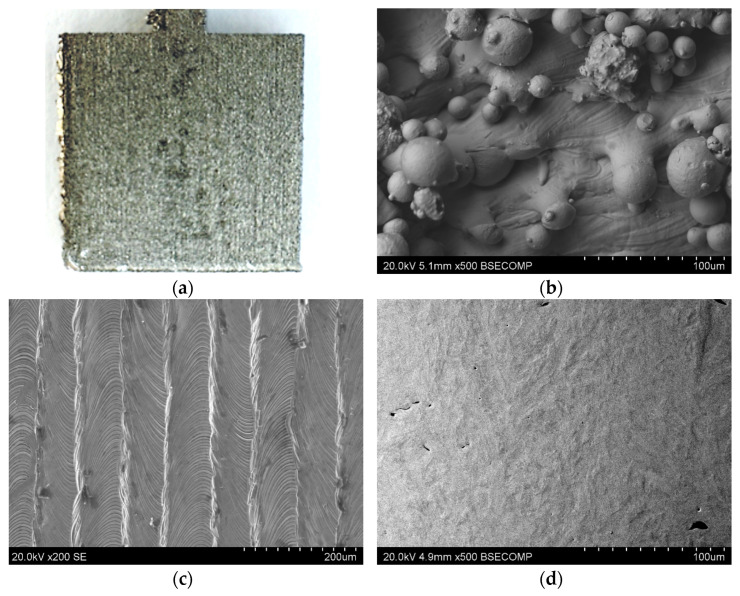
Photo of the unpolished sample before testing, (**a**) macroscopic, (**b**) sample surface (front) ×500, (**c**) sample surface (top) ×200, (**d**) sample surface metallographic cross-section (front) ×500. (SEM).

The samples made of M300 steel after the 3D-printing process were rough, which was visible to the naked eye. The lines characteristic of 3D printing, where the material was bonded, were also visible. The roughness of the material can be seen in both the macroscopic and microscopic photos presented in Figure 4. Additionally, the microscopic photo clearly shows the surface of the sample and the recesses and elevations on it.

The results of the EDS test conducted for the sample after 3D printing without polishing and not subjected to corrosion tests are presented in Table 3. The test noted a high Fe content of 79.99% by weight in the tested micro-area. Additionally, elements such as nickel, molybdenum, and titanium were detected, which are found in the standard composition of M300 steel. Additionally, sulfur was detected, which is a contaminant, and the permissible amount in this material is 0.01%.

The samples that were subjected to mechanical polishing were smooth and had no visible or palpable irregularities on their surface.

### 3.2. Potentiostatic Test

**Figure 5:**
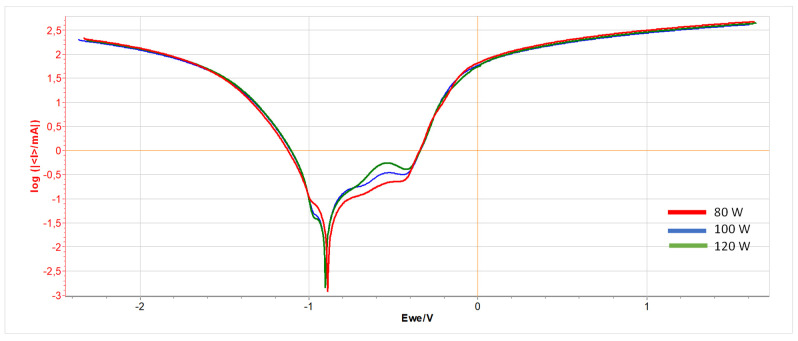
Potentiodynamic test graph for unpolished samples printed with power: 80 W (red line), 100 W (blue line), and 120 W (green line).

The results of the potentiostatic test conducted for unpolished samples printed with different laser power (80 W, 100 W, and 120 W) are presented in Figure 5. Table 4 presents the electrochemical parameters obtained during the test.

From the graph in Figure 5, one can see a similar course of the test curve for individual samples differing in the laser power used. The graph for the material printed with power of 120 W differs from the others with a noticeable slight rise at the value of −0.5 Ew e/V.

Table 4 shows that samples printed with lower laser power are characterized by higher corrosion resistance. At the same time, with increasing laser power, the rate of corrosion progress decreases.

**Figure 6:**
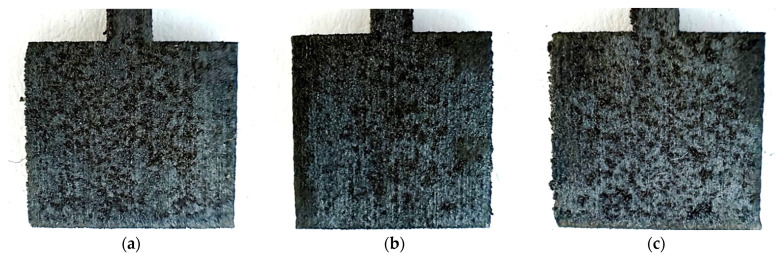
Photos of unpolished samples after the potentiostatic test, printed with laser power: (**a**) 80 W, (**b**) 100 W, and (**c**) 120 W.

The surface of the materials subjected to the test became dark and, in some places, rusty inclusions can be seen. These changes were observed for all unpolished samples printed with different powers, and are shown in Figure 6.

**Figure 7:**
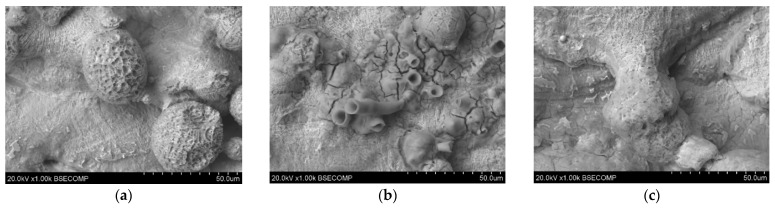
Microscopic images of unpolished samples printed with laser power: (**a**) 80 W (1000× magnification), (**b**) 100 W (1000× magnification), and (**c**) 120 W (1000× magnification).

Microscopic photos showing the surface microstructure of the samples after the corrosion test are presented in Figure 7. At a magnification of 500×, traces of corrosion can be seen on the surface irregularities. Cracks and strong signs of corrosion can be seen on the material grains. Additionally, the surfaces on the samples printed with higher laser power reveal fewer irregularities.

The results of the EDS tests carried out for the above-mentioned samples are presented in Table 5. The photos in Figure 7 show fragments covered with a rusty coating. Depending on the location where the EDS test was performed, you can see the presence of larger amounts of O, as well as a lower Fe content than in the sample that was not tested. This indicates the formation of a corrosion product, which is iron oxide. The test also detected elements such as C, Ni, Mo, Al, and Ti, which are present in the chemical composition of this material. The carbon detected in the test is a contaminant in this material.

**Figure 8:**
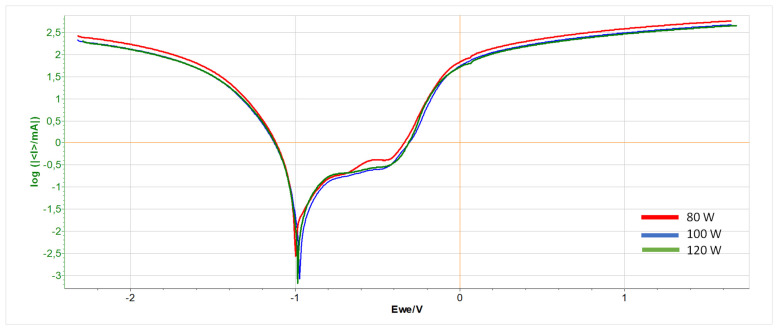
Potentiodynamic test graph for polished samples printed with power: 80 W (blue line), 100 W (green line), and 120 W (red line).

The results of the potentiodynamic test conducted for polished samples are presented in Figure 8 and in Table 6, which presents the electrochemical parameters obtained during the test.

A similar course of the test curve for individual samples can be seen from the graph in Figure 6. They differ in the vertices of the curve which, for the 120 W sample, is significantly higher than the others and, for the 80 W sample, is shifted to the right. Additionally, a slight elevation of the graph for the 120 W sample can be seen.

A significant difference in the value of the corrosion voltage can be seen from Table 5. It is 53.41 mV between the 80 W and 120 W samples. Samples printed with lower laser power are characterized by better corrosion resistance. In the case of the corrosion current, we notice different values. The corrosion process is the slowest in the 100 W sample, while the fastest in the one printed with a laser power of 120 W.

**Figure 9:**
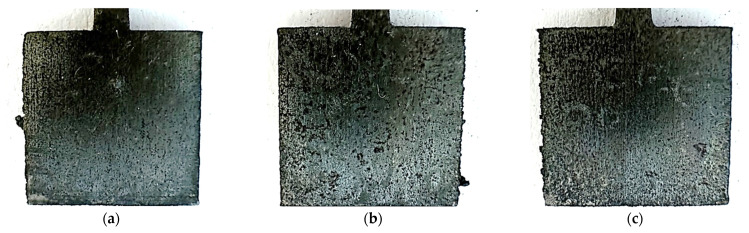
Photos of polished samples after the potentiodynamic test, printed with a laser power of: (**a**) 80 W, (**b**) 100 W, and (**c**) 120 W.

In the case of polished samples, their surface also became dark after the potentiodynamic test. In places, brown rusty discolorations can be seen. The surface also became noticeably rougher than the sample that was not tested. These changes were observed for all samples subjected to the potentiodynamic test. The surfaces of the samples are shown in Figure 9.

**Figure 10:**
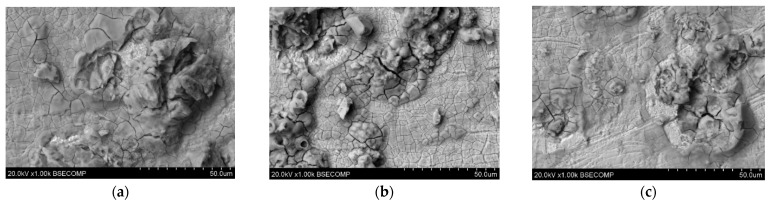
Microscopic photos of polished samples printed with a laser power of: (**a**) 80 W (magnification 1000×), (**b**) 100 W (magnification 1000×), and (**c**) 120 W (magnification 1000×).

Figure 10 presents microscopic photos of polished samples after the test. There are traces of pitting corrosion on the surface, but no significant unevenness of the material surface can be detected.

The EDS test results are shown in Table 7. The photos of the samples in Figure 8 show rusty inclusions. Large amounts of O together with the Fe indicate the presence of iron oxide. In addition, small amounts of Na and Cl were detected, which are probably a residue from the test in the NaCl solution. Small amounts of sulfur, which is a contaminant in this material, were also detected. Additionally, the EDS test showed the presence of Mo and Ti on the tested surface in each of the samples and Al in one of the samples.

**Figure 11:**
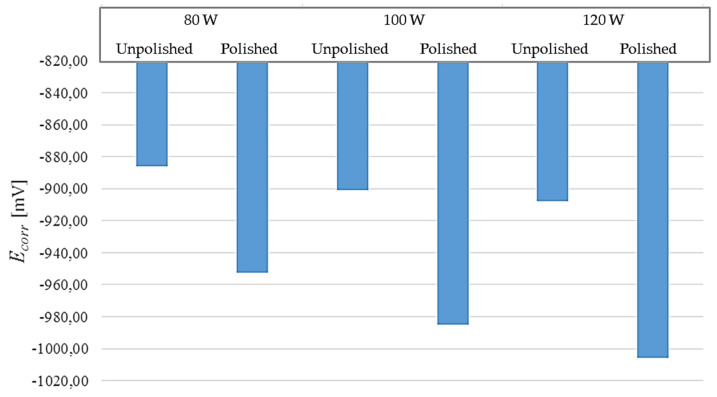
Comparison of corrosion potentials obtained during the potentiodynamic test in relation to the surface condition.

**Figure 12:**
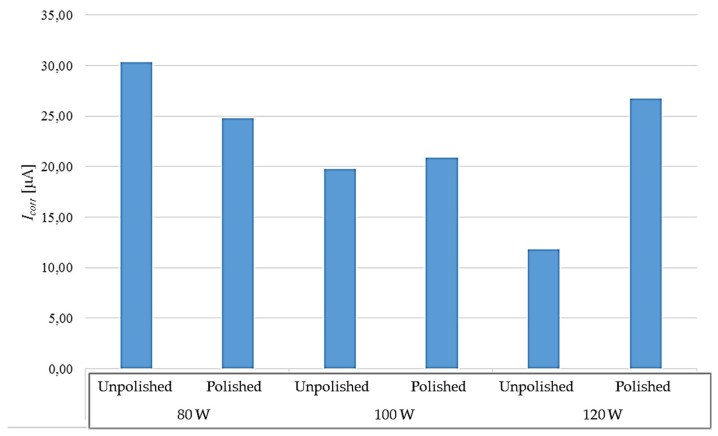
Comparison of corrosion currents obtained during the potentiodynamic test in relation to the surface condition.

In Table 8, the results of the tests carried out for polished and unpolished samples are compared. These results are also presented in the form of graphs in Figure 11 and Figure 12.

The results of the test show that better corrosion resistance is characteristic of samples that were not polished after the 3D-printing process. For each laser power, the corrosion potential was lower for unpolished samples.

The situation is different for the results of the corrosion current value, where no repeatable pattern can be observed. The higher this parameter, the faster the corrosion process progresses. In the case of samples printed with 80 W power, the corrosion process will be slower for polished materials. On the other hand, in the case of samples printed with 100 W laser power and 120 W power, unpolished materials will corrode slower. It is worth paying attention to the 120 W samples, where a significant difference of 14.86 μA can be seen in the value of this parameter.

### 3.3. Long-Term Corrosion Test

**Figure 13:**
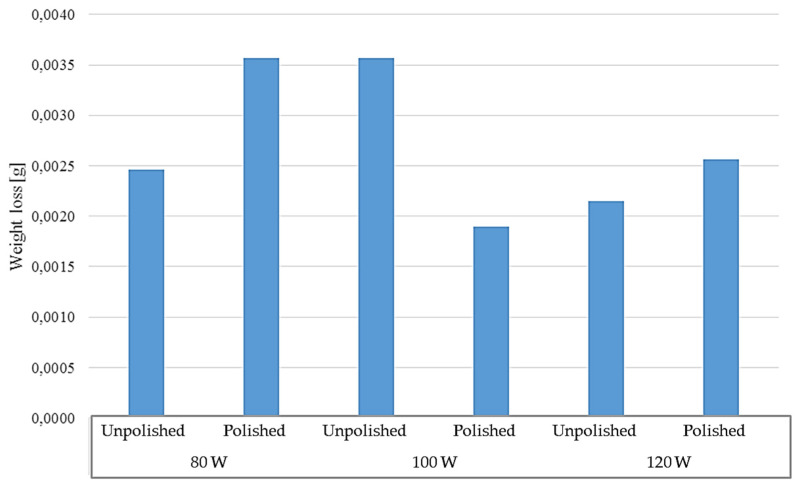
Weight loss of samples tested at room temperature.

The long-term test was carried out in two temperatures: room and elevated (40 °C), for polished and unpolished samples, printed with different laser powers: 80 W, 100 W, and 120 W. The test lasted seven days. The solution in which the samples were tested at room temperature became cloudy and rusty in color. The test results for the samples tested at room temperature are presented in Table 9 and Table 10 and in Figure 13.

**Figure 14:**
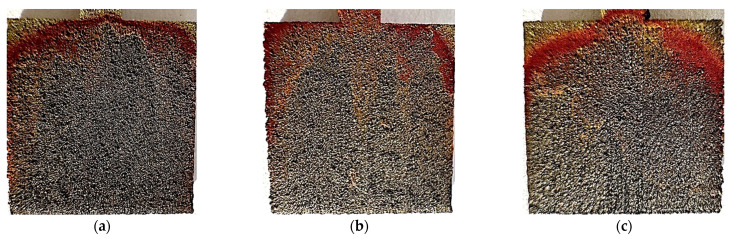
Photos after testing at room temperature for unpolished samples made with laser power: (**a**) 80 W, (**b**) 100 W, and (**c**) 120 W.

**Figure 15:**
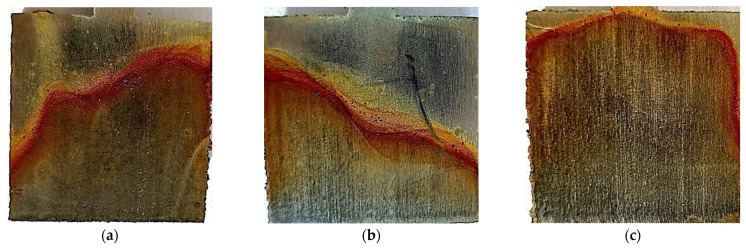
Photos after testing at room temperature for polished samples made with laser power: (**a**) 80 W, (**b**) 100 W, and (**c**) 120 W.

The test results indicate a greater weight loss of the material in the case of unpolished samples. The effect on the weight loss is also noticeable when comparing the laser power with which the 3D printing was performed. In the case of unpolished samples, the lowest value of 0.0025 g was observed for samples printed with a power of 80 W. Samples printed at 100 W and 120 W achieved the same mass loss value of 0.0036 g. Among the elements that were polished, it can be seen that the 120 W samples had the same mass loss value as the unpolished 80 W samples, i.e., 0.0025 g. This value decreases for samples polished and printed at 100 W and 80 W, and is 0.0021 g and 0.0019 g, respectively. Figure 14 and Figure 15 show photos of unpolished and polished samples after the test. A significant darkening of the material can be observed on both unpolished and polished samples. In both cases, rust-colored deposits occur. In the case of unpolished samples, they occur at the top of the samples. In the case of samples polished at 80 W and 100 W, the rusty coating appears at the mid-height while, at 120 W, it appears at the top of the sample, as in the case of unpolished samples. In Figure 14, showing the polished samples, one can see the appearance of visible material bonding lines during the 3D-printing process.

**Figure 16:**
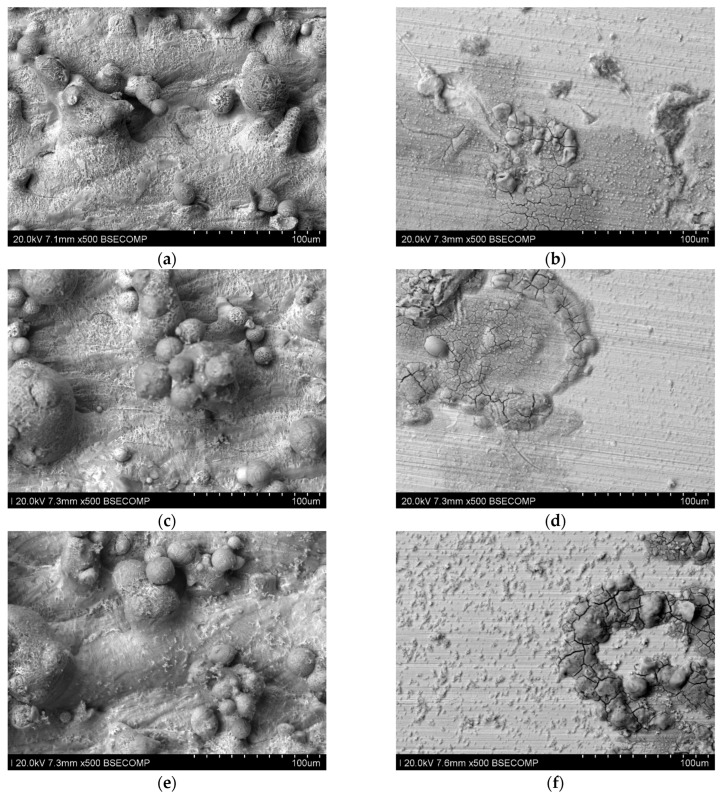
Microscopic photos of samples tested at room temperature (seven days) printed with different laser power. Unpolished surface: (**a**) 80 W (500× magnification), (**b**) 100 W (500× magnification), and (**c**) 120 W (500× magnification); polished surface: (**d**) 80 W (500× magnification), (**e**) 100 W (500× magnification), and (**f**) 120 W (500× magnification).

Figure 16 presents microscopic photos taken on samples after long-term testing at room temperature, for polished and unpolished samples. In the photos of unpolished samples, you can see unevenness and traces of corrosion occurring at the grain tops. Polished samples do not have significant unevenness, but you can see places where stronger corrosion of the material occurred.

The EDS test results are presented in Table 11. In two of the six tested samples, the element S was detected, which is a contaminant for M300 steel. In Figure 13 and Figure 14, which present photos of the samples after the test, rusty deposits can be seen. The EDS test confirmed the occurrence of iron oxide through the presence of high O and Fe content in the tested micro-areas.

**Figure 17:**
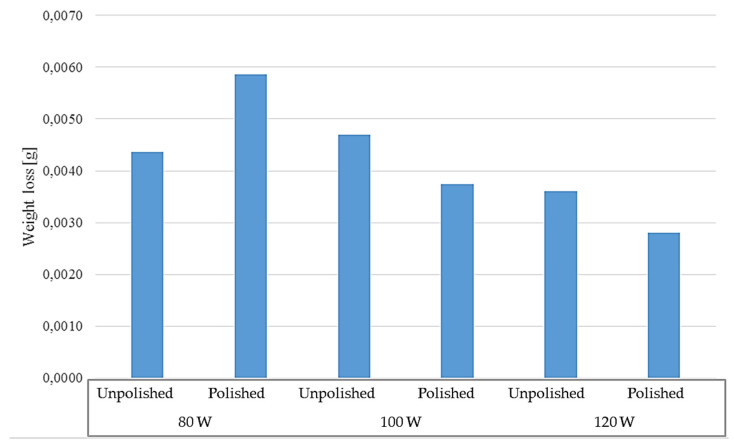
Weight loss of samples tested at elevated temperature.

The results of the long-term test conducted at an elevated temperature of 40 °C are presented in Table 12 and Table 13 and in Figure 17. Testing at an elevated temperature of 40 °C allowed for the simulation of the operating temperature of elements made of M300 steel in technical fluids reaching similar temperatures during operation.

The test results show that unpolished samples had a greater weight loss during the test than polished samples. The highest weight loss was observed for unpolished samples printed with 100 W power, which was 0.0059 g, while the lowest was for polished samples printed with 120 W power, which was 0.0028 g. In the case of polished samples, a decrease in weight loss during the test period can be observed with the increase in laser power used for their production. However, there is no such relationship for materials not subjected to the polishing process. The highest weight loss was observed for samples with 100 W power, then for 120 W, and the lowest weight loss was observed for 80 W samples.

**Figure 18:**
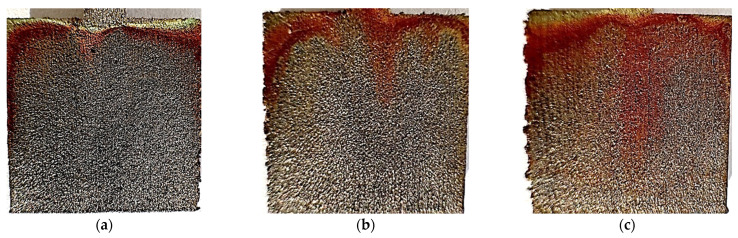
Photos after the test conducted at 40 °C for unpolished samples made with laser power: (**a**) 80 W, (**b**) 100 W, and (**c**) 120 W.

**Figure 19:**
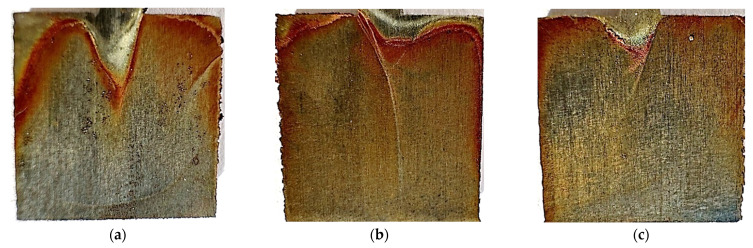
Photos after the test conducted at 40 °C for polished samples made with laser power: (**a**) 80 W, (**b**) 100 W, and (**c**) 120 W.

Figure 18 and Figure 19 show photos of unpolished and polished samples after the test at 40 °C. Significant darkening of the material can be observed on both unpolished and polished samples. On all samples, a rusty coating can be seen, concentrated at the top of the samples. The unpolished sample printed at 120 W has significantly more coating than the others. In the polished samples, pitting that did not occur before the test can be seen, especially visible in the element printed at 80 W. The edges of the samples also became irregular and uneven.

**Figure 20:**
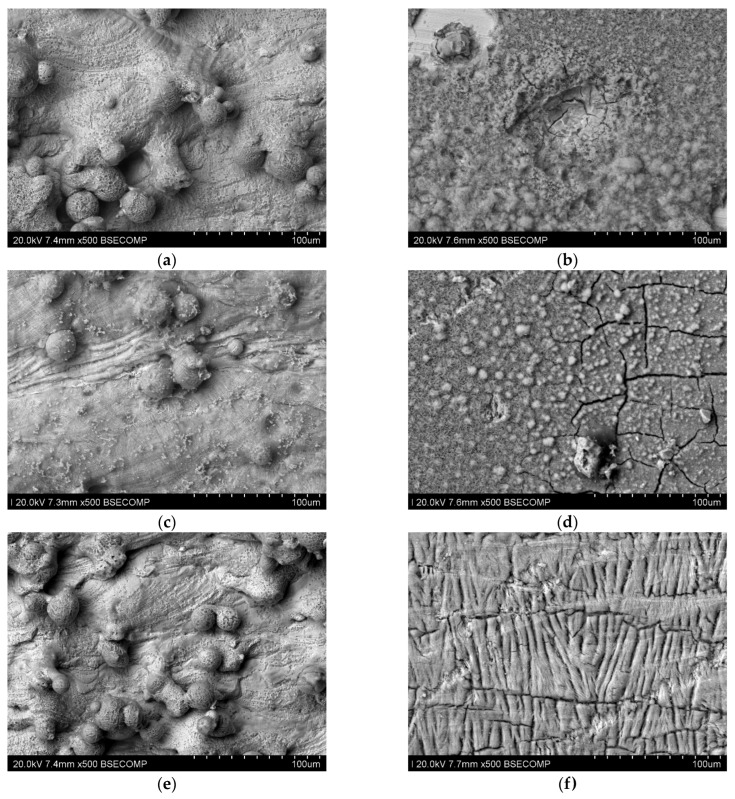
Microscopic photos of samples tested at 40 °C (seven days) printed with different laser power. Unpolished surface: (**a**) 80 W (500× magnification), (**b**) 100 W (500× magnification), and (**c**) 120 W (500× magnification); polished surface: (**d**) 80 W (500× magnification), (**e**) 100 W (500× magnification), and (**f**) 120 W (500× magnification).

Figure 20 presents microscopic photos taken on samples after the long-term test at 40 °C for polished and unpolished samples. For unpolished samples, significant surface unevenness can be observed which is, however, smaller than that occurring after testing at room temperature. In the case of polished samples, it can be seen that the surface, despite the occurrence of several surface defects, still does not have significant unevenness.

In the photo of the polished sample printed with 80 W power, you can see inclusions that are a possible effect of corrosive action.

The EDS test results for the above samples are presented in Table 14. Comparing the test results with the photos of the samples from Figure 18 and Figure 19, one can see the presence of a large amount of corrosion product, which is iron oxide, which is manifested by the presence of a rusty color on the samples and the presence of large amounts of O compared to Fe in the EDS test. These values are variable, depending on the place where the test was performed. Additionally, elements that are impurities in M300 steel, such as S and C, were detected, as well as elements occurring in the composition of this steel, i.e., Mo, Ti, Ni, and Al.

**Figure 21:**
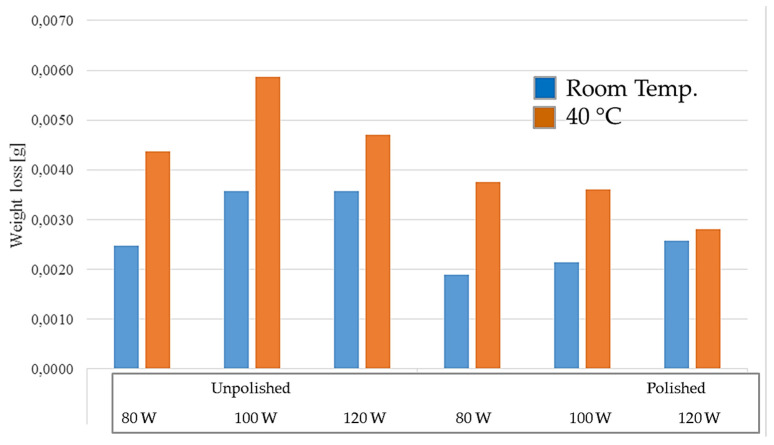
Long-term test results comparison graph for room temperature and 40 °C.

Table 15 and Table 16 present the results of long-term tests conducted for polished and unpolished samples at room temperature and 40 °C. The results are also presented in the form of a graph in Figure 21. The results obtained during the study show that unpolished samples located in a corrosive environment at elevated temperature are most susceptible to corrosion. The best resistance was demonstrated by polished 80 W and 100 W samples located at room temperature. The difference in mass loss between the highest result recorded for the unpolished sample printed at 100 W and located at an elevated temperature and the sample with the lowest loss, i.e., polished, printed at 80 W, located at room temperature, was 0.0040 g.

The calculations carried out in Table 16 allowed the obtainment of the results of the long-term impact of the corrosive environment on elements made of M300 material printed with different power, at individual operating temperatures.

## 4. Discussion

The conducted studies show the influence of laser power in the 3D-metal-printing process on the corrosion resistance of the obtained elements. The results regarding the influence of power were obtained both in the potentiodynamic study and the long-term study. The potentiodynamic study showed a negative influence of increasing the laser power in the 3D-printing process on corrosion resistance. In the case of both unpolished and polished samples, corrosion resistance decreases with the use of higher power, which was shown by the comparison of the corrosion potential for the elements subjected to the study. The corrosion current obtained in the same study shows the speed at which the corrosion process occurs in the material. The higher it is, the faster the corrosion occurs. Analysis of the results for this parameter also shows differences in corrosion resistance depending on the laser power used. In the case of unpolished samples, the rate of corrosion progress decreases with the increase of the laser power used to produce the elements. The same is true for polished samples printed with 80 W and 100 W. The value of the corrosion current decreases with the increase of the laser power. However, polished samples printed with 120 W have a higher value of this parameter than 80 W and 100 W samples and are characterized by the fastest corrosion process among polished samples subjected to potentiodynamic testing. In the long-term study, the weight loss of the samples was measured after a seven-day test, showing the effect of corrosion on the given elements. Two different behaviors were observed for polished samples. In the case of elements subjected to testing at room temperature, with the increase in the laser power used, an increase in mass loss was observed. A different trend occurred in the case of the test performed at an elevated temperature. In this case, with the increase in laser power, the mass loss after the test decreased. For unpolished samples, in the case of the test at room temperature, an initial increase in mass loss was observed with the increase in the laser power used, but the mass loss was exactly the same for the 100 W and 120 W samples. The test at an elevated temperature gave results that did not clearly indicate the effect of laser power on the mass loss, which was the lowest for the 80 W samples and the highest for the 100 W samples. In this case, the elements printed with a laser power of 120 W were in the middle of the results. The obtained results do not allow for an unambiguous determination of the effect of the applied laser power on corrosion. Based on the potentiodynamic test, it can be stated that increasing the laser power has a negative effect on the corrosion resistance of the printed material. These results were confirmed during the long-term test at room temperature. However, in the case of elevated temperature, the results in one case indicate a decrease in the impact of corrosion with increasing laser power, and in the other case do not clearly indicate a trend related to laser power. Additionally, the potentiostatic study shows that, in the case of unpolished materials, increasing laser power causes a slower rate of corrosion. In the case of polished samples, such a trend also occurs, except for the 120 W samples, in which, according to the study, corrosion will occur faster.

The impact of the surface finish of the elements was examined during both the potentiostatic and long-term study. During the potentiostatic study, significant differences were noted in the values of corrosion potentials responsible for the corrosion resistance of the elements, depending on the differences in the surface condition of the elements. Unpolished samples were characterized by a significantly higher value of the above parameter than polished samples, which theoretically means better corrosion resistance in the case of unpolished elements. The value of the corrosion current obtained during the study indicates different values in relation to the surface condition. In the case of samples printed with a power of 100 W and 120 W, the value of this parameter was higher for polished samples, indicating a faster corrosion process. A different situation occurred at a power of 80 W, where unpolished materials showed a higher value of the corrosion current, translating into a faster corrosion rate. The long-term study showed a significant effect of the surface condition on the corrosion effect on the samples. In each case, polished samples recorded a smaller mass loss than their corresponding unpolished samples.

The analysis of the results of the conducted studies is not unequivocal. On the one hand, the potentiostatic study showed poorer corrosion resistance in polished samples, as well as a faster occurrence of this process in most polished samples. However, the long-term study, showing the practical effect of the corrosion phenomenon, gave different results. In the course of this study, in each case, a smaller mass loss of the material was confirmed in the case of polished samples compared to unpolished ones.

The effect of temperature on the corrosion processes of the samples was examined during the long-term study. In each case, the mass loss was greater for samples tested at an elevated temperature. The differences are significant in many cases, reaching almost twice the mass loss for samples at elevated temperatures, e.g., in the case of polished samples printed with a power of 80 W. The only samples for which the difference is small and amounted to only 0.0002 g of mass loss are polished samples printed with a power of 120 W. Several mechanisms may be responsible for the significant increase in the impact of the corrosion phenomenon. With increased temperature, faster ion diffusion occurs. This process is associated with temperature, which provides more kinetic energy to the molecules. This increases the mobility of ions and their ability to move in the electrolyte. Also, with increased temperature, the density and viscosity of the corrosive liquid change. In liquids with lower viscosity, the movement of ions is faster, which can increase the rate of corrosion by accelerating electrochemical processes. Additionally, increasing the temperature of the liquid facilitates the penetration of the liquid between the lines of the sintered material, which can significantly affect the corrosion behavior of 3D-printed elements. The development of the results showed in which cases the greatest risk of corrosion should be expected, and also when it will occur the fastest. The results of the long-term study allowed us to learn about the real impact of the corrosive environment on the samples subjected to the test. The calculations performed additionally allowed us to estimate the weight loss of elements made of M300 steel using the 3D-printing method after a year of staying in a corrosive environment. The results may suggest the need to properly protect the elements made in the manner described in this work against the impact of corrosive environments, to change the material or production method, or to use cooling liquids with anti-corrosion additives during processing, due to the possible occurrence of corrosion in M300 steel. Considering the specialist applications of the above material, the possible impact of corrosion should be seriously considered when using the steel. The analysis of the test results suggests the protection of M300 steel components produced by the DMLS method for the case of operation in corrosive environments.

## 5. Conclusions

Based on the conducted research, the following conclusions were drawn:

- M300 steel printed using the DMLS method is susceptible to corrosion in corrosive environments;
- The laser power used during 3D printing affects the corrosion resistance of the material and the rate of corrosion;
- Increasing the laser power usually causes an increase in the corrosion phenomenon in elements located in a corrosive environment;
- The surface condition of the elements affects the corrosion phenomenon;
- Unpolished elements, after the 3D-printing process, despite higher corrosion resistance, lose mass to a greater extent in a corrosive environment;
- At room temperature, with the increase in the laser power used, an increase in mass loss is observed;
- At an elevated temperature, with the increase in laser power, the mass loss after the test decreases;
- Polished elements are less susceptible to corrosion than unpolished elements;
- The surface microstructure of the elements is degraded after contact with a corrosive environment;
- Elements made of M300 steel using the DMLS 3D printing method should be appropriately protected against the effects of a corrosive environment.

## Figures and Tables

**Table 1:** Chemical composition of maraging M300 steel (wt.%), mean value of three measurements.

Ni	Co	Mo	Ti	Mn	Si	C	P	S	Fe
18.50	9.25	4.75	0.90	0.10	0.15	0.015	0.005	0.005	balance

**Table 2:** Selected parameters of the 3D-printing process.

Parameter	Value
Laser powder	80 W
	100 W
	120 W
Laser spot size	100 µm
Laser speed	600 mm/s
Scanning path spacing	100 µm
Laser beam scanning strategy	Changing the scanning angle of each of the individual powder layers by 67° 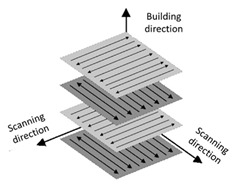
Layer thickness	30 µm

**Table 3:** Composition of the starting material in the tested micro-area.

Element	C-K	Si-K	Ti-K	Fe-K	Ni-K	Mo-L
Atomic %	2.25	0.08	1.42	79.99	14.68	1.57

**Table 4:** Electrochemical parameters obtained during potentiostatic test for unpolished samples.

Laser Power	*E_corr_*, mV	*I_corr_*, μA
80 W	−885.82	30.35
100 W	−901.16	19.74
120 W	−907.97	11.86

**Table 5:** Chemical composition of micro-area corrosion samples for samples printed with different laser powers.

	Atomic %									
Laser Power	C-K	O-K	Si-K	Al-K	Ca-K	Ti-K	Fe-K	Co-K	Ni-K	Mo-L
80 W	4.32	37.56	0.07	0.96	0.07	1.06	28.40	4.67	7.00	1.88
100 W	3.00	48.25	0.10	0.74	0.02	-	38.99	3.45	5.38	0.08
120 W	4.22	43.65	0.01	0.19	0.06	0.11	37.87	1.73	3.57	0.23

**Table 6:** Electrochemical parameters obtained during the potentiodynamic test for polished samples.

Laser Power	*E_corr_,* mV	*I_corr_*, μA
80 W	−952.55	24.81
100 W	−985.10	20.89
120 W	−1005.96	26.72

**Table 7:** Chemical composition of micro-area corrosion samples for samples printed with different laser powers.

	Atomic %									
Laser Power	C-K	O-K	Si-K	Al-K	Ca-K	Ti-K	Fe-K	Co-K	Ni-K	Mo-L
80 W	7.16	43.56	0.28	0.92	0.07	0.48	36.25	3.79	6.03	1.53
100 W	5.35	45.25	0.60	0.76	-	0.08	39.60	2.00	5.27	1.09
120 W	15.98	33.76	0.11	0.17	-	0.04	43.85	1.67	4.17	0.16

**Table 8:** Comparison of parameters obtained in the potentiodynamic test for both types of samples.

	Laser Power					
Parameter	80 W		100 W		120 W	
	Unpolished	Polished	Unpolished	Polished	Unpolished	Polished
*E_corr_*, mV	−885.82	−952.55	−901.16	−985.10	−907.97	−1005.96
*I_corr_*, μA	30.35	24.81	19.74	20.89	11.86	26.72

**Table 9:** Weight loss of samples tested at room temperature.

Weight Loss After 7 Days						
	Unpolished			Polished		
Laser power	80 W	100 W	120 W	80 W	100 W	120 W
Weight loss [g]	0.0025	0.0036	0.0036	0.0019	0.0021	0.0026

**Table 10:** Corrosion rate of samples at room temperature.

Corrosion Rate						
	Unpolished			Polished		
Laser power	80 W	100 W	120 W	80 W	100 W	120 W
Corrosion rate [(g/cm^2^)/year]	0.0459	0.0664	0.0664	0.0354	0.0400	0.0478

**Table 11:** Chemical composition of samples subjected to long-term corrosion at room temperature in the tested micro-area.

		Atomic %										
	Laser Power	C-K	O-K	Si-K	Al-K	Ca-K	Ti-K	Fe-K	Co-K	Ni-K	Mo-L	S-K
Unpolished	80 W	4.84	45.91	0.13	0.55	0.05	0.48	50.15	2.00	3.63	1.28	0.9
	100 W	3.81	45.95	0.20	0.03	-	0.69	40.11	2.16	4.57	2.13	-
	120 W	5.98	38.76	0.13	0.14	-	0.90	43.85	0.77	2.29	3.08	-
Polished	80 W	3.75	34.14	0.16	0.24	0.02	0.88	44.45	5.57	9.73	1.12	0.18
	100 W	4.83	46.26	0.06	0.34	0.03	0.69	39.54	2.45	4.34	1.47	-
	120 W	3.73	40.36	0.04	0.36	-	0.71	46.50	2.49	4.34	1.47	-

**Table 12:** Weight loss of samples tested at elevated temperature.

Weight Loss After 7 Days						
	Unpolished			Polished		
Laser power	80 W	100 W	120 W	80 W	100 W	120 W
Weight loss [g]	0.0044	0.0059	0.0047	0.0037	0.0036	0.0028

**Table 13:** Corrosion rate of samples at elevated temperature.

Corrosion Rate						
	Unpolished			Polished		
Laser power	80 W	100 W	120 W	80 W	100 W	120 W
Corrosion rate [(g/cm^2^)/year]	0.0813	0.1093	0.0875	0.0698	0.0670	0.0521

**Table 14:** Chemical composition of samples subjected to long-term corrosion at 40 °C in the tested micro-area.

		Atomic %										
	Laser Power	C-K	O-K	Si-K	Al-K	Ca-K	Ti-K	Fe-K	Co-K	Ni-K	Mo-L	S-K
Unpolished	80 W	8.57	38.80	0.26	0.34	-	1.77	37.47	3.86	6.25	2.51	0.17
	100 W	8.81	40.95	0.30	0.26	-	7.69	43.11	2.16	4.57	7.69	-
	120 W	4.92	36.71	0.19	0.11	-	3.90	44.97	2.70	2.09	3.24	-
Polished	80 W	8.25	42.14		0.07	0.01	2.56	37.59	2.77	6.32	0.14	0.16
	100 W	8.84	39.14	0.03	0.06	0.04	0.29	45.29	2.01	3.78	0.57	-
	120 W	7.61	45.46	0.11	0.9	-	0.33	40.11	2.33	3.34	0.50	0.07

**Table 15:** Summary of long-term test results for room temperature and 40 °C.

Mass Loss After 7 Days						
	Unpolished			Polished		
	80 W	100 W	120 W	80 W	100 W	120 W
Room temperature	0.0025	0.0036	0.0036	0.0019	0.0021	0.0026
Temperature 40 °C	0.0044	0.0059	0.0047	0.0037	0.0036	0.0028

**Table 16:** Corrosion rate comparison of samples at room temperature and 40 °C.

Corrosion Rate [(g/cm^2^)/year]						
	Unpolished			Polished		
	80 W	100 W	120 W	80 W	100 W	120 W
Room temperature	0.0459	0.0664	0.0664	0.0354	0.0400	0.0478
Temperature 40 °C	0.0813	0.1093	0.0875	0.0698	0.0670	0.0521

## Data Availability

The original contributions presented in this study are included in the article. Further inquiries can be directed to the corresponding author.
